# Depression and Anxiety in Older Adults: A Comparison Between Online Convenience and Conventional Representative Sampling

**DOI:** 10.1155/da/2522358

**Published:** 2025-07-01

**Authors:** Hana Georgi, Eva Dragomirecká, Zuzana Tichá, Josef Mana

**Affiliations:** ^1^Prague College of Psychosocial Studies, Prague, Czech Republic; ^2^Department of Psychology, Faculty of Arts, Charles University, Prague, Czech Republic

**Keywords:** affective mental health, online survey, recruitment, sampling bias

## Abstract

**Background:** Depression and anxiety are among the most common mental health issues of older populations, and as such they are frequently monitored covariates. The possibilities for collecting research data has grown with the recent emergence of user-friendly online survey platforms. However, to what extent the populations of older persons who participate in such research are similar to the general population remains unclear. We investigated the affective health of an open online sample of older adults (65+) in contrast to a representative randomised in-person interview sample.

**Methods:** The surveys were conducted in 2021 during the COVID-19 pandemic in the Czech Republic after the second wave of anti-COVID-19 vaccination. The online sample (*N* = 389) was recruited via the Internet. Participants of the in-person study (*N* = 633) were randomly approached according to quotas for representativeness. The administered questionnaires included a health status self-report, the Geriatric Depression Scale (GDS) and the Geriatric Anxiety Inventory – Short form (GAI-SF).

**Results:** Online participants reported better mental and general health; that is, they reported fewer depressive and anxiety symptoms than the randomised representative sample. In both samples, women showed higher levels of anxiety than men. Subjective general health was associated with mental health. In the randomised representative sample, in contrast to the online sample, the level of depression increased significantly with age. The open non-randomised calls for participants attracted a higher percentage of women and people with higher education than are in the general older population.

**Conclusions:** Older research volunteers recruited online can be expected to be subjectively healthier and to differ largely from the general population in their sociodemographic characteristics.

## 1. Introduction

Depression and anxiety are among the most prevalent mental health issues of older age (aside from dementia), with frequent comorbidity of the two. Diagnosed either separately or in conjunction, depression and anxiety exert a devastating toll on quality of life, sapping individuals' vitality and dimming their perspectives [1, 2]. Depression and anxiety are negatively associated with cognitive performance and form a risk factor for cognitive decline and progression to dementia [3–7]. For this reason, depression in particular is often controlled for in neuropsychological studies, for example [8, 9]. In the realm of research, a plethora of self-assessment instruments has been employed to gauge the prevalence and severity of depression and anxiety in older adults. These tools involve face-to-face or telephone interviews, self-administered questionnaires filled out in controlled settings as well as online surveys [10].

In psychology, there is a tradition of using both population-based random representative and convenience samples. The comparative evaluation of these two sampling methods has yielded mixed results, with some studies demonstrating close similarities in findings [11], while others reveal significant discrepancies [12]. This ongoing debate surrounding generalisability and sampling bias has become a recurring theme [13, 14]. Convenience sampling, often criticised for its limitations in generalisability due to potential selection bias, has several distinct advantages. It is typically less expensive, more readily implemented and expedites data collection from willing participants [15] compared with population-based random representative samples [16], which are widely regarded as the 'gold standard' for ensuring the large-scale generalisability of findings. The main drawback of convenience samples is that they are drawn from a readily available segment of the population, meaning they are unlikely to be a true reflection of the broader population of interest, that is, the external validity and the replicability are limited, and the risk of selection and volunteer bias is increased [15, 17]. Despite its drawbacks, convenience sampling has found broad application in normative and psychometric studies based on neuropsychological tests [18, 19] or psychological questionnaires [20, 21]. To mitigate the inherent limitations of convenience sampling, researchers have implemented strategies, such as enforcing age, gender, and education quotas. This approach has garnered recognition as an acceptable alternative to probability sample surveys [22]. Post-stratification methods can be used to generalise beyond the current sample [23]. However, to conduct a valid post-stratification analysis, the ways in which convenience samples differ from the population of interests need to be identified first.

The rapid evolution of communication technologies has opened up new avenues for online recruitment of convenience samples through crowdsourcing platforms like Amazon Mechanical Turk [24], social media such as Facebook [25] and specialised research volunteer databases [26]. Beyond recruitment, researchers are increasingly employing Internet communication to collect data, including data from older persons [27], or to deliver psychotherapeutic interventions [28]. Not long ago, it was thought that older persons cannot be reached by online surveys, but that has changed [29]. In the Czech Republic, too, the number of older persons who use the Internet is growing [30], and as of 2022, about one-half of the senior population (65+ years old) had Internet access [31]. Consequently, online surveys specifically designed for older adults are gaining traction [32, 33]. While the online collection of data has opened up new possibilities, the quality of the data obtained and differences in sample characteristics remain a matter of ongoing debate [34–36].

The main aim of this study was to examine the affective health of the Czech online older population and contrast it with the general older population. This constitutes a unique opportunity to get a comprehensive view of the similarities and disparities between results attained using two distinct recruitment and data-collection approaches involving non-random samples of older adults within the same timeframe during a pandemic: an open online survey (ONLINE) and a representative paper-based, face-to-face interview survey (REPRE).

The comparisons presented here aspire to provide novel perspectives on the affective health profiles of online older adults and on the generalisability of findings derived from online surveys.

Both our studies (ONLINE and REPRE) obtained approvals from the relevant institutional review boards of the Prague College of Psychosocial Studies (no. 6/2021) and the Faculty of Arts, Charles University (no. UKFF/477759/2021). Both studies were conducted in accordance with the Declaration of Helsinki as amended in 2013 [37]. All participants digitally confirmed (ONLINE) or signed (REPRE) an informed consent. Participants received no remuneration. The compared studies can be viewed as being at a low risk of bias, with ≥ 3 points according to the modified Newcastle–Ottawa risk of bias scoring guide which assesses the urban/rural representativeness of the sample, sample size > 200 or convenience sample, described non-respondents, ascertainment of depression by a valid instrument, and the quality of reporting descriptive statistics [38].

## 2. Methods

### 2.1. Participants and Recruitment Strategies

The ONLINE survey was open from July until September 2021, that is, during the second year of the COVID-19 pandemic, between the second and third wave of available anti-COVID-19 vaccination. It was advertised via Facebook and on the websites of the Czech-Moravian Psychological Society and the Prague College of Psychosocial Studies, with further participants recruited by the snowball method. This strategy resulted in a non-random convenience sample. In the end, we had 546 responses (537 of which were unique). For this study, we excluded 98 datasets from persons aged 60–64 and included only data from participants aged 65+. Another 50 respondents were excluded due to incomplete responses to the questionnaires. The final dataset therefore included 389 persons.

The survey was advertised as a study aimed at standardising psychological measures of the well-being of older adults. The invitation to participate specifically targeted individuals aged 60 and above. All participants who opted to receive results and provided their email address were sent a personalised email containing their raw scores for the Geriatric Depression Scale (GDS-15) and Geriatric Anxiety Inventory – Short form (GAI-SF) and a commentary explaining the comparison of their scores to the standard cut-off point. If the results indicated a potential risk of mental health concerns (scores higher than standard cut-offs), the email included a recommendation to consult a physician, particularly if the respondent had any sleep-related problems.

The REPRE data were collected face-to-face by a specialised agency, INRES-SONES, and its 130 trained agents as part of a large interdisciplinary project KREAS of the Faculty of Arts (Charles University, Prague) in the fall of 2021, also between the second and third wave of available anti-COVID-19 vaccination. Participants were recruited by agency staff who randomly contacted households in selected municipalities from the list of the Czech Statistical Office (CZSO). Participants were interviewed at home. The agents followed preset quotas for representativeness [39] in terms of gender, age, education, region, and municipality size. Eligible were persons who had no known dementia diagnosis, consented to participation and were at least 65 years old. All in all, 720 eligible persons were invited; 12.1% refused for various reasons, with women somewhat more reluctant to participate (12.4% women and 11.6% men refused participation). On the other hand, the most easily recruitable group were the youngest women eligible for participation (those aged 65–74), with only 8.9% rejections. The final sample consisted of 633 eligible participants.

### 2.2. Questionnaires

In both the ONLINE and the REPRE sample, we collected the same sociodemographic data (level of education, occupation, marital status, and type of residence) and administered several specific questionnaires. General health was assessed using the Short Form Health Questionnaire (SF-12). Its first item (SF1) is a valid single-question self-assessment of health on a 5-point scale (excellent–very good–good–fair–poor) [40]. The GDS (or its 15-item version, GDS-15) is a self-report instrument designed to screen for depressive symptoms in older adults. The usual optimal cut-off score for detecting depressive symptomatology is 5/6, with a reported sensitivity of 80.5% and a specificity of 75.0% [41, 42]. We then administered the short form of the GAI-SF, which was designed as a self-report instrument to detect anxiety symptoms common in older age. The GAI-SF includes 5 Yes/No items and the cut-off score used is 2/3, with a sensitivity of 75% and a specificity of 87% for Generalised Anxiety Disorder [43]. The GAI-SF was selected instead of the GAI full-length version due to its time efficiency while maintaining psychometric quality, which allowed other methods to be included in the surveys.

All these measures (SF-12, GDS-15, and GAI-SF) are used in Czech clinical practice and research, and Czech language mutations are available [44, 45]. Higher scores on the GDS-15 and GAI-SF indicate more pronounced symptoms and therefore a higher risk of depression and anxiety disorder, respectively.  Table 1 provides an overview of the characteristics of the final ONLINE and REPRE samples.

## 3. Analyses

**S**tatistical analyses were performed using the jamovi 1.6.23 freeware and R software for statistical computing (version 4.3.3.). The sociodemographic characteristics, as well as the GDS-15 and GAI-SF scores of the ONLINE and REPRE samples were compared using the Mann–Whitney *U*-test for continuous and the *χ*^2^ test for categorical variables. We created a binomial variable—‘mental health'—derived from the GDS-15 and GAI-SF scores (no mental issues if neither score exceeded the standard cut of 5/6 for the GDS-15 and 2/3 for the GAI-SF). The numbers of persons who exceeded the cut-off scores for suspected depression [41] and anxiety [43] in both samples were compared using the *χ*^2^ test. To evaluate the effect of sampling modality (ONLINE vs. REPRE) on mental health outcomes that is not attributable to demographic differences, we conducted a set of regression analyses with mental health variables as outcomes, the sampling modality as a predictor of interest and relevant sociodemographic variables (age, gender, education level, and health status in SF1) as covariates^1^. We used linear regression for continuous outcomes (GDS-15 and GAI-SF raw scores) and logistic regression for binary outcomes (suspected depression and anxiety). For all analyses, the level of significance was set at 0.05. The code used for data analysis is available at https://github.com/josefmana/AffectiveSeniors.git.

## 4. Results

For all pairwise comparisons of the samples (Table 1). Compared to the REPRE sample, the ONLINE sample was on average younger, had higher proportion of women, lower proportion of people with lower education level, more city residents, and better self-reported health status (SF1). Moreover, the ONLINE sample had lower mean scores of GDS-15 and GAI-SF and lower proportion of people with suspected depression or anxiety. The GAI-SF and the GDS-15 significantly correlated positively in both the REPRE (rho = 0.611, *p*  < 0.001) and the ONLINE (rho = 0.537, *p*  < 0.001) samples.

**Figure 1:**
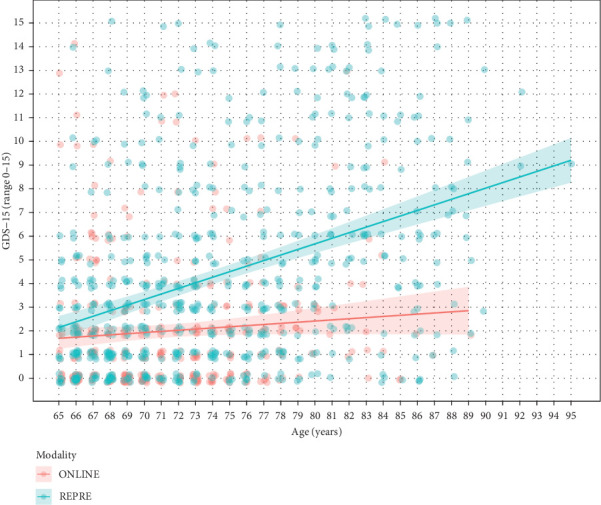
Total GDS-15 score as a function of age in the ONLINE and REPRE samples. *Note*: The plot shows data (scattered points) and their linear prediction by age separately for ONLINE and REPRE samples.

The results of regression analyses are presented in Table 2. We observed a statistically significant interaction effect between the sampling modality (ONLINE vs. REPRE) and person's age on depression symptoms; both the raw GDS-15 score and the binary suspected depression. Specifically, depressive symptoms were higher in older people compared to younger people in the REPRE sample but not in the ONLINE sample (Figure 1). The expected GDS-15 score was similar between the youngest age groups and increasingly higher in the REPRE compared to the ONLINE sample with increasing age.

After accounting for the effect of sociodemographic variables, there was remaining statistically significant difference between REPRE and ONLINE samples in either depression or anxiety outcomes. Gender had an effect on anxiety measures whereby women scored higher than men. Both higher depressive symptoms and higher anxiety symptoms were associated with higher SF1 scores. Neither one of these effects statistically significantly differed between REPRE and ONLINE samples.

Finally, because the raw GDS-15 and GAI-SF data exhibited some degree of both ceiling and floor effects, we additionally fitted a series of Bayesian regression models that allow for directly modelling these data structures. For parsimony's sake, these analyses are presented in the Supporting information. Overall, the models fitted raw GDS-15 and GAI-SF data substantially more closely than classical linear regression and their results aligned well with results presented in Table 2.

## 5. Discussion

The main aim of this study was to examine the affective health of the Czech online older population and contrast it with the general older population. We also aimed to investigate potential differences that may be associated with two frequently used study designs in social science research of older adults (65+): An online survey with a convenience sample recruited via a website, social media, or invitation based on previous online participation, and a representative quota-randomised study. We found that the sample of online older adults in total reported significantly lower levels of anxiety and depressive symptomatology than the general older population. Online sample showed minimum levels of depression and anxiety very similar to previous Czech normative studies of GDS-15 and GAI-SF, which were also based on convenience samples [44, 45].

In both samples, women reported greater levels of anxiety than men. Our data do not support an effect of gender on levels of depression. Literature is generally in agreement that anxiety is more prevalent among women up to an old age [46], and thus both REPRE and ONLINE samples fit in [45, 46]. Reports on depression are not that decisive, yet a systematic review found female gender among risk factors of depression in late life [47]. Our results do not support that. The main difference between the samples in depression was driven by age. In REPRE oldest persons reported higher levels of depression, while ONLINE reported very low levels also in the oldest groups. We did not find a similar pattern for anxiety, though.

Our results show that online methods can deliver a large-enough sample of older persons: We managed to collect data from several hundred older adults up to the age of 89. The sample size clearly demonstrates that many older persons are active online, can be accessed via online media and are willing to participate in psychological surveys that include questions related to their mental health. Analyses showed that the online survey attracted more women, more persons with higher education and in subjectively better health, more inhabitants of larger cities and fewer persons whose (former) occupation was manual than the representative survey did. Looking at the overall picture, we can conclude that the online strategy attracted an 'elite' group of seniors: People who reported significantly fewer mental health issues and better health status than the general population (REPRE sample). The self-selection and undercoverage biases stand out.

The ONLINE sample's age distribution reflected the reported Internet use by older adults and their age distribution according to the CZSO [31]. Two-thirds of our ONLINE sample were among the youngest of our respondents (aged 65–74). In the Czech population, 61% of the young-old seniors (65–74 years of age) but only 30% of the older (75+) group have access to and use the Internet.

The positive significant correlation between anxiety and depression symptoms in our study corresponds to the findings of other studies. For example, Egbert et al. [48] reported a significant association of self-reported health, depression, and anxiety in a large sample of 111,225 persons from 58 countries within a broad age range of 18–110 years (15,326 of them were older adults 55+) who filled in an online survey during the COVID-19 pandemic in 2020 and were recruited mainly through the Internet, including online social media. That study also showed a protective effect of education in older adults: Persons with higher education typically reported fewer symptoms of depression and anxiety. A meta-analysis identified low education as one of the factors associated with depression in old age [47], while the reports pertaining to association of anxiety and educational attainment are not that clear [1]. Our ONLINE sample included disproportionately more persons with higher education than the REPRE sample (92.8% vs. 48.2%), therefore, the different levels of education could have been an underlying cause for the found differences in levels of depression and anxiety. However, the regression analyses did not confirm an effect of education neither in the REPRE nor the ONLINE sample on the mentioned mental health parameters.

It is well documented that good health is associated with lower levels of depressive symptoms in older persons. Halaschek-Wiener et al. [49] showed in a sample of 480 healthy, long-lived individuals who were recruited at the age of 85+, had no history of cancer, cardiovascular disease, diabetes, dementia, or major pulmonary disease, and were recruited in 2004–2007 as part of the Super-Seniors study, that they had a minimal GDS-15 total score (*M* = 1.5; SD = 1.8). Our results are in line with theirs. Both the REPRE and ONLINE samples showed a strong association of better subjective health (SF1) with fewer depressive and anxiety symptoms.

Besides the effect of age on depression, we hypothesise the reason for the lower mental health in the compared studies is the random strategy of recruitment in REPRE, which contrasted with the self-selection and resulting convenience sampling of the normative and current ONLINE studies. Only the REPRE sample can be considered fully representative because it was designed to match the demographics of the Czech population, and the selection was randomised. Also, we suggest to consider the results as proof of research projects attracting mentally healthier and more resilient volunteers regardless the pandemic who proactively join in as participants. In other words, the random recruitment (probability representative sample) yielded the most accurate picture of the state of mental health among older Czech adults during the second year of the pandemic.

### 5.1. Limitation

The ONLINE design did not enable participants to ask for clarification of individual items, which may have played a role in their answers and subsequently led to a measurement error [50]. This is one of the most significant caveats of online surveys. We tried to minimise the risk of poor comprehensibility by having four researchers go through the preliminary version of the survey with two older persons (76 and 89 years old with secondary education) and adjusting the language where possible. The standard wording of the specific scales was not adapted because we had to ensure compatibility with other studies.

The main limitation is that while both REPRE and ONLINE were realised in 2021 after the second wave of vaccination, which meant a breaking point in the pandemic, they differed in seasons. ONLINE was open in the summer, and REPRE was collected in the fall. The seasons are sometimes considered a risk factor for lower mood, yet a recent systematic review showed there was no convincing support in the literature for that [51]. Older persons seem to be more resilient towards the seasonal effects on mood [52]. Besides seasonality, higher scores of depression and anxiety in REPRE may be partially due to another wave of the COVID-19 infections. This difference would not be expected to be so prominent, though, as the initial increase of mental health issues in the first year was significant in all age groups [53], but it was shown to decline with time and the older generations were surprisingly resilient in comparison to young people (e.g. [54]).

Neither the ONLINE nor the REPRE studies included any test of cognitive status. Therefore, both the ONLINE and REPRE datasets may include data from persons with a cognitive impairment. Given the demands of either participating in the ONLINE or organisation of the REPRE assessment and the length of the assessment, we believe that, including only the fully completed data means there is a very limited possibility that persons with a pronounced cognitive impairment or dementia could be included in the two datasets.

In terms of ethnicity, Czech society is highly homogeneous, with an absolute majority of people being Caucasian. The Czech Republic has some relatively small historical minorities of people of Roma and Vietnamese origin maintaining their culture, but only a very low number of people of African or, for instance, Hispanic descent. Our studies targeted rather uniformly Czech speakers because cultural specifics could be considered another source of variability that might complicate the comparability of the results. However, in view of the growing numbers of non-first-generation adults from ethnic minority groups, future research should focus also on them.

## 6. Conclusion

Older persons who volunteer in online surveys report significantly lower levels of depression and anxiety than the general population. Technological advances enable the online collection of data in the social sciences, including psychology, and in some cases also normative or psychometric studies [55, 56]. The COVID-19 pandemic and restrictions associated with it dramatically accelerated the amount of online activities, and what used to be optional or even non-existent before suddenly became obligatory or the only way to do particular things. This applied mainly to learning, office work, some medical consultations, or even treatment (e-health), but also to social science research [57, 58]. Online recruitment and data collection have many advantages, such as cost-effectiveness and speed, but also certain limitations including self-selection bias and questionable external validity [15, 59]. Our study confirmed the feasibility of recruiting large numbers of older persons for online psychological surveys and yielded some characteristics of typical participants. We also described certain differences between an online convenience sample and a representative face-to-face sample: It seems, for instance, that online surveys attract the ageing 'super-elite', that is, persons who enjoy above-average levels of physical and mental health. Our analyses suggest that convenience samples in general include more 'elite' volunteers who report less anxiety and fewer depression symptoms than the general population. Such 'elite' groups deserve further study because their lifestyle and other factors may point to ways of better ageing. On the other hand, if we want to get a true picture of reality, we need to use better recruitment strategies to attract research participants who may not age as well and have limited access to communication channels that are common for the 'elite'. Last but not least, we listed some likely possible causes of differences between our findings and the normative studies. We can conclude that when using convenience sampling, both in-person and online, researchers ought to be aware of the need to describe their recruitment strategy and data collection process in detail to highlight the sources of potential bias [17], especially when they are seeking to elicit general, internationally valid facts. This will lead to a better reproducibility of studies.

## Figures and Tables

**Table 1:** Sociodemographic characteristics and basic comparisons of the ONLINE and REPRE samples.

Variable	Category	ONLINE		REPRE		Stat.	*p*	Effect size
		*N*	%	*N*	%			
*N*	—	389	—	633	—	—	—	—
Age	—	*M* = 71.3 SD = 4.97	—	*M* = 74.0 SD = 6.90	—	*U* = 97,426	<0.001	0.209
Age range	—	65–89 years	—	65–95 years	—	—	—	—
Age range	80+	96	24.7	258	40.8	*χ* ^2^ = 27.5	<0.001	0.164
Gender	Women	308	79.2	366	57.8	*χ* ^2^ = 48.9	<0.001	0.219
Education	Lower	28	7.2	328	51.8	*χ* ^2^ = 211	<0.001	0.455
Residence	City	179	46.0	144	22.7	*χ* ^2^ = 60.34	<0.001	0.243
Status	Living with partner/spouse	194	49.9	353	55.8	*χ* ^2^ = 3.37	0.067	0.057
Occupation (former)	Manual or mainly manual	41	10.5	469	74.1	*χ* ^2^ = 389	<0.001	0.617
Health	SF1 score	*M* = 2.43 Mdn = 2 SD = 0.76	—	*M* = 3.29 Mdn = 3 SD = 0.89	—	*U* = 59,393	<0.001	0.518
GDS-15	Total score	*M* = 1.99 Mdn = 1 SD = 2.74	—	*M* = 4.25 Mdn = 3 SD = 4.05	—	*U* = 76,456	<0.001	0.379
GDS-15	Suspected depression (GDS-15 > 5)	42	10.8	187	29.5	*χ* ^2^ = 48.7	<0.001	0.218
GAI-SF	Total score	*M* = 0.85 Mdn = 0 SD = 1.51	—	*M* = 1.40 Mdn = 1 SD = 1.67	—	*U* = 96,349	<0.001	0.217
GAI-SF	Suspected anxiety (GDS-SF > 2)	58	14.9	152	24.0	*χ* ^2^ = 12.21	<0.001	0.109
Mental health	No anxiety, no depression	316	81.2	401	63.3	*χ* ^2^ = 36.8	<0.001	0.190

*Note:* Lower education—either primary or secondary education without final state exams; city—population over 100 thousand inhabitants. Reported effect size for Mann–Whitney *U*: Rank biserial correlation, and effect size for *χ*^2^: Cramer's *V*.

**Table 2:** Results of regression analyses aimed to estimate the adjusted effect of sampling modality (ONLINE vs. REPRE) on mental health markers.

Model term	Depression symptoms				Anxiety symptoms			
	GDS-15		Sus. depression		GAI-SF		Sus. anxiety	
	*F* ratio	*p*	*χ* ^2^	*p*	*F* ratio	*p*	*χ* ^2^	*p*
Modality	1.864	0.172	0.241	0.623	0.623	0.430	0.287	0.592
Age	**15.478**	**<0.001**	3.350	0.067	0.445	0.505	0.025	0.875
Gender	0.000	0.990	0.006	0.938	**13.643**	**<0.001**	**6.445**	**0.011**
Education	0.624	0.430	0.117	0.733	0.017	0.896	0.077	0.781
SF1	**231.704**	**<0.001**	**78.598**	**<0.001**	**103.753**	**<0.001**	**53.823**	**<0.001**
Modality:age	**10.361**	0.001	**7.609**	**0.006**	2.451	0.118	3.305	0.069
Modality:gender	0.001	0.975	0.061	0.805	1.214	0.271	1.053	0.305
Modality:education	0.339	0.560	1.558	0.212	1.119	0.290	0.649	0.421
Modality:SF1	1.829	0.177	0.644	0.422	0.539	0.463	0.000	1.000

*Note:* Modality: sampling procedure (REPRE vs. ONLINE). *F* ratio: *F* statistic with 1 and 1012 degree of freedom. *χ*^2^: *χ*^2^ statistic with 1 degree of freedom. Statistics associated with model terms that reached the threshold for statistical significance are printed in bold.

## Data Availability

The raw data from ONLINE and REPRE are available from the corresponding author upon request.

## Endnotes

^1^To ensure the inferred effects are not due to moderating effects, interaction between modality and all covariates was included in the models and interpreted first.

## Supporting Information

Supporting Information Supporting information presents Bayesian regression models that were developed to directly model the distribution of the raw GDS-15 and GAI-SF data, which exhibited both ceiling and floor effects.
