# Supplementary material for: Depression and Anxiety in Older Adults: A Comparison Between Online Convenience and Conventional Representative Sampling
Source: Depress Anxiety. 2025 Jul 1;2025:2522358. doi: 10.1155/da/2522358 (PMC12237557; doi:10.1155/da/2522358)
Supplement: Supporting Information — presents Bayesian regression models that were developed to directly model the distribution of the raw GDS-15 and GAI-SF data, which exhibited both ceiling and floor effects. [file 2522358.f1.docx]

Supplementary Material for Depression and Anxiety in Older Adults: A comparison between Online Convenience and Conventional Representative Sampling

Hana Georgi

Eva Dragomirecká

Josef Mana

Zuzana Tichá

As evidenced in [Figure 1](#bookmark=id.ljjzt2ozbo1d), the data distribution of both GAI-SF and GDS-15 show distributions that are likely not well reproduced by Gaussian distribution. Indeed, [Figure 2](#bookmark=id.1cjddzelduwz) shows that the least squares linear regression implemented in the lm() function in R that was used for analyses in the main text, misfits data distribution at several places. Notably, the floor and ceiling effects are not reproduced by linear regression. To see whether these data structures could have severely bias our results, in this Supplementary material we fit a set of Bayesian models that directly address floor and ceiling effects. All analyses were computed in R R (version 4.3.3) (R Core Team 2024), models were fitted using the *brms* package (Bürkner 2017), their posterior predictions evaluated using the *bayesplot* package (Gabry and Mahr 2024) and their posterior distributions summarised using the *bayestestR* package (Makowski, Ben-Shachar, and Lüdecke 2019).

| 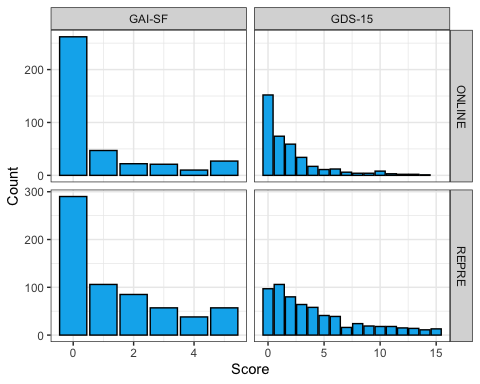  *Figure 1: Bar plots of data distribution for GDS-15 and GAI-SF in REPRE and ONLINE samples.* |
| --- |
| 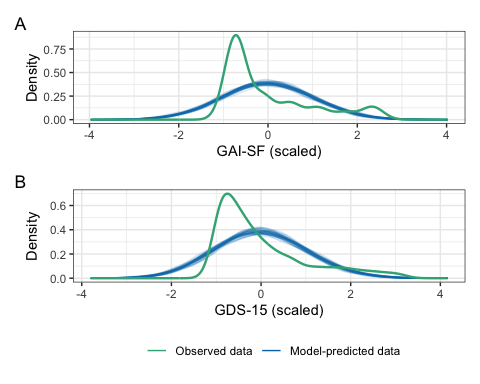  *Figure 2: Posterior predictive checks of models implied by the linear regressions reported in the main text for GAI-SF (A) and GDS-15 (B) raw scores (scaled by sample’s means and standard deviations).* |

# Count Models

One of the reasons why linear regression fails to reproduce data from GDS-15 and GAI-SF is that although the scales consist of count data, linear regression fits them via a continuous Gaussian error distribution. Possible remedy is use of a distribution for count data - in this section we evaluate binomial and zero-inflated binomial regressions.

As shown by [Figure 3](#bookmark=id.3a3lriqao25x) and [Figure 4](#bookmark=id.7iqqz2ic99uc), the zero-binomial models showed better alignment with data compared to simple binomial models. This is especially aparent when looking at model prediction of GDS-15 and GAI-SF of the full data set ([Figure 3](#bookmark=id.3a3lriqao25x)). Whereas the binomial model (left panels) misses the peak of GDS-15 distribution and overestimates probability of non-zero low scores for GAI-SF, the zero-inflated binomial model (right panels) successfully reproduces most features of the data distributions. The zero-inflated binomial model accounts well for the floor effect but not the ceiling effect of GAI-SF. It also provides predictions of similar quality for ONLINE and REPRE samples for GDS-15 but much better predictions for the REPRE compared to the ONLINE sample for GAI-SF ([Figure 4](#bookmark=id.7iqqz2ic99uc)). Overall, the zero-inflated binomial models reproduced data better than binomial models and will thus be retained for further analysis.

| 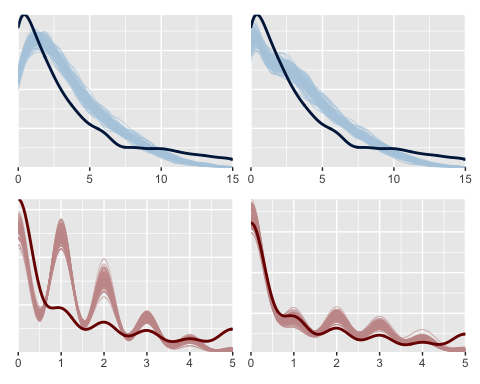  *Figure 3: Posterior predictive check of the count models with respect to the whole data set. Top row contains GDS-15 (blue), bottom row contains GAI-SF (red) model predictions and data. Binomial models are presented on the left, zero-inflated binomial models on the right.* |
| --- |
| 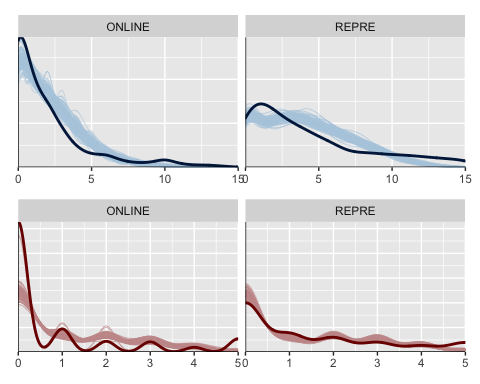  *Figure 4: Posterior predictive check of the count models separately for the ONLINE and REPRE samples. Top row contains GDS-15 (blue), bottom row contains GAI-SF (red) model predictions and data.* |

# Continuous Models

Albeit the data are count, not continuous variables, continuous distributions, such as the Gaussian, can still provide a good representation of their distribution. In this section, we evaluate censored versions of Gaussian and Student distributions to predict GDS-15 and GAI-SF. Censoring allows for a direct modelling option of the ceiling and floor effects. We evaluate both, the Gaussian as well as Student-t distribution because the latter can account for potential heavy tails caused by outliers.

As censored Gaussian and censored Student-t provide predictions almost identical in thei major features ([Figure 5](#bookmark=id.gtzkjo3nutbx)), we will focus on the former only. For GAI-SF, the censored Gaussian model was able to account for both ceiling and floor effectc giving it a slight edge against the zero-inflated binomial model discussed in the previous section. For GDS-15, it account for the floor effect more effectively than the zero-inflated binomial model, however, the censored Gaussian model did not reproduce the rest of the data, especially vey low but non-zero score, as effectively. There were no substantial differences in the censored Gaussian predictions for ONLINE versus REPRE samples ([Figure 6](#bookmark=id.uzeahwsckv5n)).

| 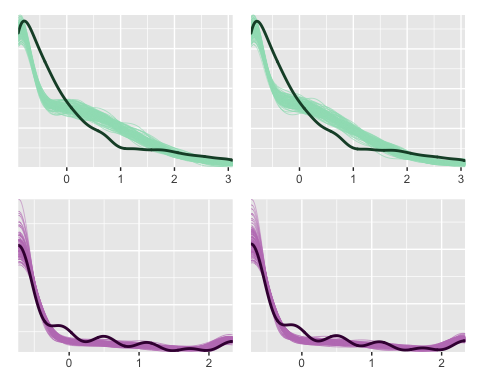  *Figure 5: Posterior predictive check of the continuous models with respect to the whole data set. Top row contains GDS-15 (green), bottom row contains GAI-SF (purple) model predictions and data. Censored Gaussian models are presented on the left, censored Student models on the right.* |
| --- |
| 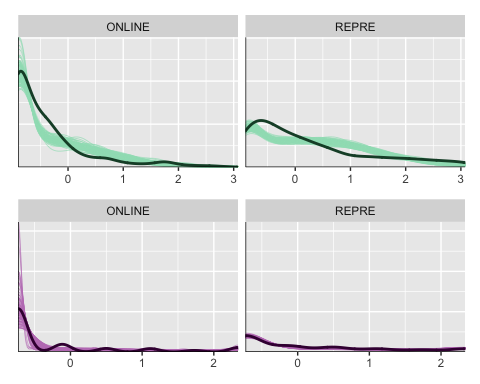  *Figure 6: Posterior predictive check of the continuous models separately for the ONLINE and REPRE samples. Top row contains GDS-15 (green), bottom row contains GAI-SF (purple) model predictions and data.* |

# Posterior Distributions

To compare the zero-inflated binomial and censored Gaussian models’ implications to the main text results, we present summary of each models’ parameters. These are summarised via their median (an estimate of the effect’s size), 95% Highest Density Posterior Interval (HDPI, a measure of the effect uncertainty), and the probability of direction (*pd*, a measure of the strength of evidence for effect existence). The results are presented in [Table 1](#bookmark=id.x9dzlqs7e9pz). The inference regarding the effect of sampling modality (REPRE vs ONLINE) remain unchanged compared to the main text.

| ***Table 1***  *Summaries of posterior distributions of model parameters of models predicting GDS-15 and GAI-SF. All continuous variables were scaled before entering into the models. Parameters of the zero-inflated binomial models are on the latent logit scale. Contrasts for binary predictors were set-up to ensure equal prior mass across all groups.*   \|  \| GDS-15 \| \| \| \| \| \| GAI-SF \| \| \| \| \| \| \| --- \| --- \| --- \| --- \| --- \| --- \| --- \| --- \| --- \| --- \| --- \| --- \| --- \| \|  \| Censored Gaussian \| \| \| Zero-Inflated Binomial \| \| \| Censored Gaussian \| \| \| Zero-Inflated Binomial \| \| \| \| Parameter \| Md \| 95% HDPI \| *pd* \| Md \| 95% HDPI \| *pd* \| Md \| 95% HDPI \| *pd* \| Md \| 95% HDPI \| *pd* \| \| Intercept \| **-0.20** \| **[-0.32, -0.08]** \| **100%** \| **-1.29** \| **[-1.37, -1.20]** \| **100%** \| **-1.19** \| **[-1.52, -0.92]** \| **100%** \| **-0.56** \| **[-0.79, -0.33]** \| **100%** \| \| Modality \| 0.09 \| [-0.07, 0.26] \| 87.4% \| 0.12 \| [0.00, 0.24] \| 97.2% \| 0.30 \| [-0.08, 0.72] \| 93.1% \| -0.00 \| [-0.32, 0.29] \| 50.3% \| \| Age \| **0.16** \| **[0.08, 0.24]** \| **100%** \| **0.13** \| **[0.07, 0.18]** \| **100%** \| 0.06 \| [-0.12, 0.24] \| 75.5% \| -0.00 \| [-0.11, 0.11] \| 50.7% \| \| Gender \| 0.02 \| [-0.09, 0.13] \| 66.1% \| -0.02 \| [-0.10, 0.07] \| 63.5% \| **0.58** \| **[0.30, 0.86]** \| **100%** \| **0.40** \| **[0.18, 0.60]** \| **100%** \| \| Education \| 0.07 \| [-0.08, 0.23] \| 80.5% \| 0.01 \| [-0.10, 0.13] \| 58.1% \| -0.10 \| [-0.49, 0.25] \| 68.8% \| -0.16 \| [-0.42, 0.11] \| 88.1% \| \| SF1 \| **0.70** \| **[0.61, 0.78]** \| **100%** \| **0.84** \| **[0.78, 0.92]** \| **100%** \| **1.03** \| **[0.82, 1.23]** \| **100%** \| **0.69** \| **[0.55, 0.84]** \| **100%** \| \| Modality:Age \| **0.11** \| **[0.01, 0.23]** \| **97.7%** \| **0.18** \| **[0.10, 0.26]** \| **100%** \| 0.10 \| [-0.17, 0.34] \| 78.0% \| **0.20** \| **[0.04, 0.37]** \| **99.4%** \| \| Modality:Gender \| -0.03 \| [-0.18, 0.12] \| 65.5% \| -0.04 \| [-0.16, 0.09] \| 73.0% \| -0.05 \| [-0.45, 0.33] \| 60.4% \| 0.01 \| [-0.28, 0.30] \| 51.4% \| \| Modality:Education \| 0.05 \| [-0.18, 0.26] \| 67.2% \| **0.17** \| **[0.00, 0.32]** \| **98.4%** \| 0.36 \| [-0.15, 0.90] \| 91.5% \| 0.33 \| [-0.06, 0.69] \| 95.2% \| \| Modality:SF1 \| -0.09 \| [-0.20, 0.05] \| 91.0% \| **-0.20** \| **[-0.29, -0.11]** \| **100%** \| -0.16 \| [-0.43, 0.12] \| 88.1% \| **-0.20** \| **[-0.40, -0.01]** \| **97.6%** \| \| *Note.* Modality, sampling procedure (REPRE vs ONLINE); GDS-15, Geriatric Depression Scale, 15-item version; GAI-SF, Geriatric Anxiety Inventory – Short Form; Md, median; HDI, Highest Density Posterior Interval; pd, probability of direction, *pd* > 97.5% (printed in **bold**) can be interpreted as equivalent to two-tailed p < 0.05. \| \| \| \| \| \| \| \| \| \| \| \| \| |
| --- | --- | --- | --- | --- | --- | --- | --- | --- | --- | --- | --- | --- | --- | --- | --- | --- | --- | --- | --- | --- | --- | --- | --- | --- | --- | --- | --- | --- | --- | --- | --- | --- | --- | --- | --- | --- | --- | --- | --- | --- | --- | --- | --- | --- | --- | --- | --- | --- | --- | --- | --- | --- | --- | --- | --- | --- | --- | --- | --- | --- | --- | --- | --- | --- | --- | --- | --- | --- | --- | --- | --- | --- | --- | --- | --- | --- | --- | --- | --- | --- | --- | --- | --- | --- | --- | --- | --- | --- | --- | --- | --- | --- | --- | --- | --- | --- | --- | --- | --- | --- | --- | --- | --- | --- | --- | --- | --- | --- | --- | --- | --- | --- | --- | --- | --- | --- | --- | --- | --- | --- | --- | --- | --- | --- | --- | --- | --- | --- | --- | --- | --- | --- | --- | --- | --- | --- | --- | --- | --- | --- | --- | --- | --- | --- | --- | --- | --- | --- | --- | --- | --- | --- | --- | --- | --- | --- | --- | --- | --- | --- | --- | --- | --- | --- | --- | --- | --- | --- | --- | --- | --- | --- | --- | --- | --- | --- | --- | --- | --- | --- | --- | --- |

# References

Bürkner, Paul-Christian. 2017. “Brms: An r Package for Bayesian Multilevel Models Using Stan” 80. <https://doi.org/10.18637/jss.v080.i01>.

Gabry, Jonah, and Tristan Mahr. 2024. “Bayesplot: Plotting for Bayesian Models.” <https://mc-stan.org/bayesplot/>.

Makowski, Dominique, Mattan S. Ben-Shachar, and Daniel Lüdecke. 2019. “bayestestR: Describing Effects and Their Uncertainty, Existence and Significance Within the Bayesian Framework.” 4: 1541. <https://doi.org/10.21105/joss.01541>.

R Core Team. 2024. *R: A Language and Environment for Statistical Computing*. Vienna, Austria: R Foundation for Statistical Computing. [https://www.R-project.org/](https://www.r-project.org/).
